# Low Household Income Increases Hospital Length of Stay and Decreases Home Discharge Rates in Lumbar Fusion

**DOI:** 10.1177/21925682241239609

**Published:** 2024-03-21

**Authors:** Ryan S. Gallagher, Ritesh Karsalia, Austin J. Borja, Emelia G. Malhotra, Maria A. Punchak, Jianbo Na, Scott D. McClintock, Neil R. Malhotra

**Affiliations:** 1 Department of Neurosurgery, 6572 Perelman School of Medicine at the University of Pennsylvania, Philadelphia, PA, USA; 2 6572 University of Pennsylvania, Philadelphia, PA, USA; 3 The West Chester Statistical Institute and Department of Mathematics, West Chester University, West Chester, PA, USA

**Keywords:** household income, length of stay, discharge disposition, lumbar fusion

## Abstract

**Study Design:**

Retrospective Matched Cohort Study.

**Objectives:**

Low median household income (MHI) has been correlated with worsened surgical outcomes, but few studies have rigorously controlled for demographic and medical factors at the patient level. This study isolates the relationship between MHI and surgical outcomes in a lumbar fusion cohort using coarsened exact matching.

**Methods:**

Patients undergoing single-level, posterior lumbar fusion at a single institution were consecutively enrolled and retrospectively analyzed (n = 4263). Zip code was cross-referenced to census data to derive MHI. Univariate regression correlated MHI to outcomes. Patients with low MHI were matched to those with high MHI based on demographic and medical factors. Outcomes evaluated included complications, length of stay, discharge disposition, 30- and 90 day readmissions, emergency department (ED) visits, reoperations, and mortality.

**Results:**

By univariate analysis, MHI was significantly associated with 30- and 90 day readmission, ED visits, reoperation, and non-home discharge, but not mortality. After exact matching (n = 270), low-income patients had higher odds of non-home discharge (OR = 2.5, *P* = .016) and higher length of stay (mean 100.2 vs 92.6, *P* = .02). There were no differences in surgical complications, ED visits, readmissions, or reoperations between matched groups.

**Conclusions:**

Low MHI was significantly associated with adverse short-term outcomes from lumbar fusion. A matched analysis controlling for confounding variables uncovered longer lengths of stay and higher rates of discharge to post-acute care (vs home) in lower MHI patients. Socioeconomic disparities affect health beyond access to care, worsen surgical outcomes, and impose costs on healthcare systems. Targeted interventions must be implemented to mitigate these disparities.

## Introduction

Social determinants of health (SDOH) such as income, wealth, education, employment, and food security have been widely studied and are known to influence health outcomes broadly on both individual and population scales.^1-6^ Literature on the effects of SDOH such as race, gender, and economics in neurosurgery is emerging, yet gaps remain in identifying how these factors specifically influence surgical outcomes and how they can be mitigated.^7^

Median household income is known to be associated with mortality and has been associated with multiple medical and surgical outcomes.^3,8-10^ In neurosurgical studies, lower median household income has been correlated to increased 30- and 90 day mortality for posterior fossa brain tumor cases,^11,12^ increased mortality in glioblastoma,^13^ and increased 90 day ED visits for supratentorial meningioma resections.^14^ Salwi et al^15^ demonstrated a higher composite socioeconomic score correlated to improved outcomes and decreased mortality in stroke.

In spine surgery specifically, Lambrecths et al implicated a correlation between household income and radiographic severity of cervical degenerative disc disease.^16^ Barrie et al^17^ found that SES was significantly correlated to increased length of stay, as well as 90 day opioid prescriptions and ER visits for all spine surgery patients in their cohort. Chan et al^18^ recently found that socioeconomic status augmented the predictive value of the Centers for Medicare and Medicaid Services Hierarchical Condition risk adjustment model for adverse outcomes such as mortality, length of stay, and non-home discharge in a large cohort undergoing lumbar fusion.

Lumbar fusion is a common spine procedure worldwide that has been increasing in frequency and utilization.^19^ Previous research has demonstrated that appropriately selected patients experience an increase in healthcare-related quality of life after spine surgery, and optimizing postoperative outcomes though risk-mitigation strategies is important for long-term improvements in these patients.^20,21^ Although household income has been shown to correlate with negative outcomes for many types of surgery, there are complex interactions with other socioeconomic and medical factors such as age, race, body mass index, and comorbidity. Prior research in spine surgery has not controlled for such factors to establish the direct impact of household income. In a widely performed surgery with good outcomes and limited complications at baseline,^22^ the correlation between household income and surgical outcomes may be due to complex interactions between 1 or more of these factors.

Building upon prior studies, this study aims to isolate any effect that Median Household Income (MHI) may have on short-term outcomes among patients undergoing single-level, posterior-only lumbar fusion at a single academic medical center. We implement a coarsened exact matching (CEM) protocol to control for patient-level factors independently related to surgical outcomes and isolate the influence of MHI. By this method, high and low-income patients are paired 1:1 by finding those who exactly match across all controlled variables, allowing the isolation of the effect of income.^23^

## Methods

### Sample Selection

**Figure 1.**
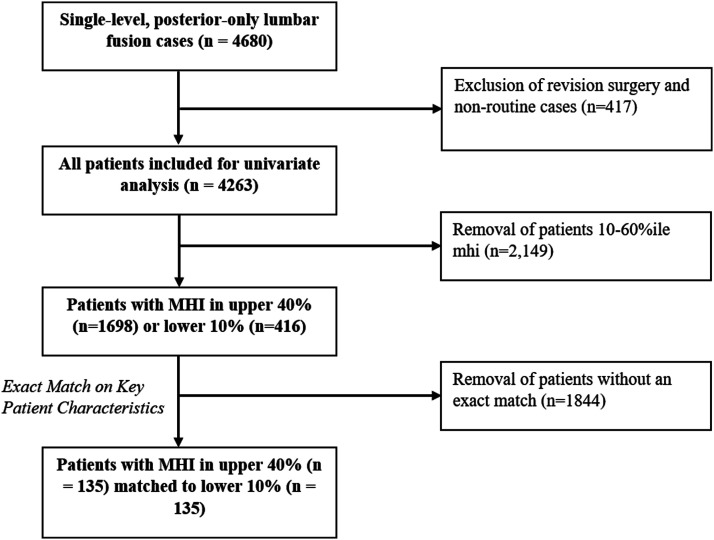
Study selection and sample size.

Consecutive adult patients undergoing non-revision, single-level posterior-only lumbar fusion surgery between 2013-2021 were enrolled in our study (Figure 1), yielding 4679 observations. This study was approved by the Institutional Review Board at the Hospital of the University of Pennsylvania (IRB 832794). Informed consent was waived as this study was considered minimal risk to patients. Our cohort was limited to include only clean wounds, general anesthesia, routine (non-urgent) inpatient admissions, and body mass index (BMI) between 10 and 70, thus equating to a total of 4263 observations.

### Data Extraction

Patient and outcome data were obtained using Epilog – a non-proprietary data acquisition system integrated with the electronic health record (EHR) to facilitate quality improvement initiatives without disrupting workflow.^24^ Patient zip-code was cross-referenced to the 2012-2016 U.S. Census Bureau 5 Year American Community report to derive MHI adjusted for inflation to US$2016. Other obtained patient characteristics included BMI, age, gender, race, American Society of Anesthesiologists (ASA) score, smoking status, prior back surgery history, CCI score, and insurance type (public vs private). Outcomes measured included surgical complication, length of stay, discharge home vs non-home, and 30- and 90- day readmissions, emergency department (ED) evaluation, reoperation, and all-cause mortality.

### Statistical Methods

Univariate logistic regression was performed to compare MHI with patient outcomes. Patients were then split into deciles of observed median household income values. Patients from the lowest decile of MHI (0-10) in our sample were paired with counterparts at the opposite end of the MHI spectrum in the highest 4 deciles (60-100) who matched exactly by gender, ASA grade, age (by decade), smoking, insurance type (private vs public), any prior surgery, prior surgery in 30 days, CCI (<4, 5-6, >7), BMI (<18.5, normal, and obese >30), and race (white/non-white). Income percentiles were intentionally selected to assess outcomes in the lowest 10th percentile of MHI to define a feasible target population for long-term risk mitigation strategies, similar to income-based risk stratification strategies seen in other medical specialties and value-based payment models.^25-27^ McNemar’s test was used to compare surgical outcomes between these 2 coarsened exact matched groups. Nonparametric testing was used to compare length of stay.

## Results

### Patient Characteristics

In our cohort comprising n = 4263 initially analyzed with univariate analysis, the median length of stay was 80 h. There were 1698 patients in the upper 40% of MHI and 416 in the lowest 10%. A total of 270 patients were matched exactly between these groups. Prior to matching, significant differences were found between patients in the highest 40% and lowest 10%ile of MHI with respect to age, gender, race, BMI, smoking status, CCI, ASA, and prior surgical history. After matching, no differences in these social, medical, and surgical factors remained between these groups (Table 1).

**Table 1.** Patient Demographic and Medical Information Before (n = 2114) and After (n = 270) Exact Matching, Grouped by Patients in the Top 40% and Bottom 10% by MHI. *Continuous Variables Were Compared via Nonparametric Tests, While Discrete Variables Were Compared by Chi-Squared or Fisher’s Exact Tests.

	Before Exact Matching			After Exact Matching		
Total	Top 40% (n = 1698)	Lowest 10% (n = 416)	*P*-value*	Top 40% (n = 135)	Lowest 10% (n = 135)	*P*-value*
Gender, n (%)						
Male	768 (45.2)	149 (35.8)	.001	50 (37)	50 (37)	1.0
Female	930 (54.8)	267 (64.2)		85 (63)	85 (63)	
Age, n (%)						
<50	229 (13.5)	83 (10)	<.001	25 (19)	25 (19)	1.0
50-60	361 (21.3)	132 (31.7)		31 (23)	31 (23)	
60-70	519 (30.6)	129 (31)		49 (36)	49 (36)	
70-80	491 (28.9)	55 (13.2)		26 (19)	26 (19)	
>80	98 (5.8)	17 (4.1)		4 (3)	4 (3)	
Insurance type, n (%)						
Private	837 (49.3)	136 (32.7)	<.001	65 (48.1)	65 (48.1)	1.0
Government	861 (50.7)	280 (67.3)		70 (51.9)	70 (51.9)	
Any lifetime surgical intervention prior to the index operation, n (%)						
No	1106 (65.1)	192 (46.2)	<.001	95 (70.4)	95 (70.4)	1.0
Yes	592 (34.9)	224 (53.9)		40 (29.6)	40 (29.6)	
Any surgical intervention 30 days prior to the index operation, n (%)						
No	1649 (97.1)	400 (96.2)	.31	134 (99)	134 (99)	1.0
Yes	49 (2.9)	16 (3.9)		1 (1)	1 (1)	
CCI score, n (%)						
Low (≤4)	1369 (80.6)	308 (74)	.002	115 (85.2)	115 (85.2)	1.0
Medium (5,6)	222 (13.1)	63 (15.1)		17 (12.6)	17 (12.6)	
High (≥7)	107 (6.3)	45 (10.8)		3 (2.2)	3 (2.2)	
Body mass index (kg/m2), mean						
<18.5	12 (.71)	4 (1)	<.001	-	-	
18.5 – 29.9	1021 (60.1)	182 (43.7)		71 (52.6)	71 (52.6)	1.0
>30.0	665 (39.1)	230 (55.3)		64 (47.4)	64 (47.4)	
Smoking status, n (%)						
Smoker	147 (8.7)	96 (23.1)	<.001	13 (9.6)	13 (9.6)	1.0
Non-smoker	1544 (90.9)	318 (76.4)		122 (90.4)	122 (90.4)	
Unknown	7 (.4)	2 (.5)		-	-	
Race						
White	1516 (89.28)	105 (25.2)	<.001	76 (56.3)	76 (56.3)	1.0
Non-white	182 (10.72)	311 (74.8)		59 (43.7)	59 (43.7)	
American society of anesthesiologists grade, mean (range)						
	2.40 (1-4)	2.50 (1-4)	<.001	2.33 (2-3)	2.33 (2-3)	1.0

### Statistical Results

**Figure 2.**
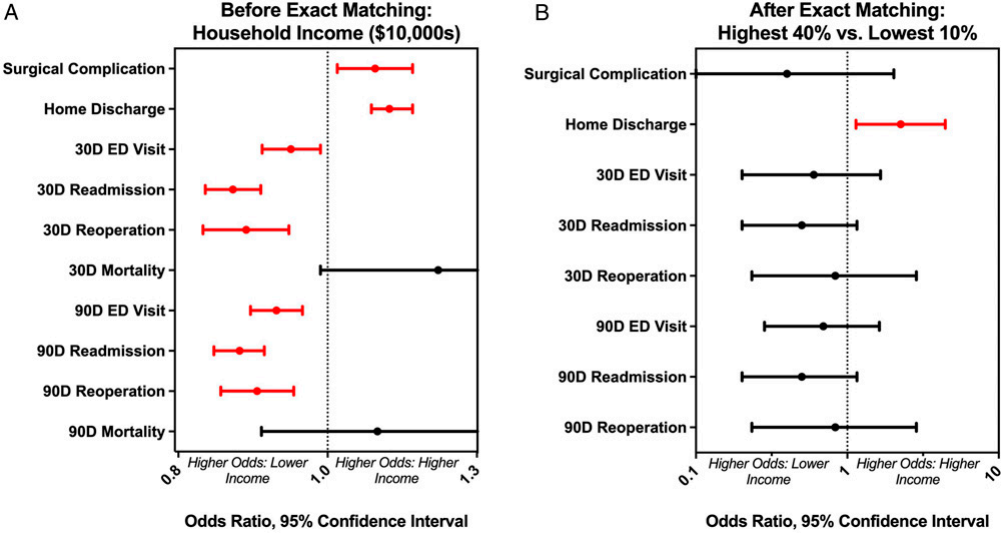
Panel A– Results of Univariate Regression. Lower MHI corresponded to significantly higher odds of non-home discharge, ED visits, readmissions, and reoperations. Higher MHI corresponded to higher odds of surgical complications. Panel B– Results of Coarsened Exact Matching. Odds ratios shown for high MHI relative to low MHI. Patients with low MHI had significantly higher odds of non-home discharge relative to otherwise exactly matched patients with high MHI.

Univariate regression analysis found that lower MHI was significantly correlated to non-home discharge and 30- and 90 day ER visits, admissions, and reoperations (Figure 2(a), Table 2). Higher MHI was significantly correlated to increased surgical complications. No significant relationship was observed between MHI and mortality at both 30- and 90 days. After coarsened exact matching, a statistically significant relationship was observed between MHI and discharge to home (OR = 2.5, *P* = .0163), indicating higher odds of non-home discharge for low-income patients (Figure 2(b), Table 2). Length of stay was higher in the low-income compared to the matched high-income group (mean 100.24 vs 92.60, *p*-.02). No significant relationship was found between MHI and ER visits, admissions, reoperations, or surgical complications between the matched groups.

**Table 2.** Results of Analyses Before (Left) and After (Right) Coarsened Exact Matching. Odds Ratios and *P*-values for Logistic Regression Reported Using MHI in US$10,000 Units. Coarsened Exact Matching Odds Ratios Comparing the Highest 40% to the Lowest 10% in MHI.

	Before Exact Matching		After Exact Matching	
	Univariate MHI (US$10,000s)		Top 40% vs Bottom 10%	
Outcome	OR (95% CI)	*P*-Value	Or (95% CI)	*P*-Value
Intraoperative complication	1.08 (1.02-1.14)	.014	.40 (.05 - 2.03)	.29
Home discharge	1.10 (1.07-1.14)	<.001	2.25 (1.14 - 4.44)	.02
30D ED visit	.95 (.90-.99)	.015	.60 (.20 - 1.66)	.33
30D readmission	.86 (.83-.90)	<.001	.50 (.20 - 1.16)	.11
30D reoperation	.88 (.83-.94)	<.001	.83 (.23 - 2.86)	.77
30D mortality	1.19 (.99-1.42)	.07	N/A	N/A
90D ED visit	.92 (.89-.96)	<.001	.69 (.28 - 1.63)	.40
90D readmission	.87 (.84-.91)	<.001	.50 (.20 - 1.16)	.11
90D reoperation	.90 (.85-.95)	<.001	.83 (.23 - 2.86)	.77
90D mortality	1.08 (.90-1.29)	.40	N/A	N/A

## Discussion

Our study extends prior work examining SES as a predictor of outcomes to lumbar spinal fusion, one of the most common procedures in neurosurgery. By univariate regression, we found a significant increase in 30-day and 90-day ED visits, readmissions, and reoperations, as well as decreased surgical complications and decreased home discharge for lower median household income for patients undergoing lumbar fusion. This is congruent with prior literature exploring the relationship between SES and surgical outcomes and with prior observations in lumbar fusion.^11,15,16,28,29^ The correlation between household income and outcomes from lumbar fusion is particularly notable given the low baseline morbidity and mortality of this procedure.^22^ As seen in Table 1, however, low MHI patients in our cohort tended more often to be female, younger, publicly insured, non-white, smokers, and have a prior history of any surgery and greater comorbidity and surgical risk measured by CCI and ASA. While higher medical risk may account for the increase in short-term resource utilization, younger age in the low MHI cohort may account for the inverse correlation seen with intraoperative complications such as unintended durotomy. Private insurance in the U.S. is often employer-based and may offer options for more extensive coverage than public insurance; that is, employment status, insurance access and coverage, and income are all correlated but may be separately driving health outcomes. These highlight the complex interactions within social determinants of health and motivate our matching analysis to isolate more direct effects of MHI on short-term lumbar fusion outcomes.

After isolating MHI from covariates by CEM, we observed that patients within the lowest ten percent of observed median household income values had over double the odds of non-home discharge compared to their higher-income counterparts and had an average hospital stay 8 hours longer. This relationship that SES relates to worsened discharge disposition has been observed in prior studies such as post-thrombectomy for stroke and arthroplasty.^30,31^ Inverse relationships between household income and length of stay have also been found in neurosurgery.^17,18^ Notably, these prior observations were gathered using multivariate regression models. By exactly matching patients one-to-one, our finding furthers this evidence to support a direct correlation between income and discharge to home. Our findings suggest that MHI may serve as a social risk adjustment tool to identify high-risk patients who may benefit from targeted interventions to reduce health inequities.

In comparison to our findings, a prior study of discharge location after joint replacement surgery generally found that lower SES was associated with lower levels of post-surgical rehabilitation care and^32^ suggested that medical or surgical complicating factors associated with lower SES may be confounding factors.^32^ In our study, the tendency for non-home discharge in lower-income patients contrasts with the lack of any other observable difference between the matched patients in surgical complications, readmissions, or reoperations. This may suggest a lack of socioeconomic support at home necessary for post-surgical rehabilitation may be responsible for our findings rather than inherent medical or surgical differences between the groups. Despite similar demographics, medical risk, and insurance coverage, low MHI may nonetheless influence the preparedness of patients for discharge after lumbar fusion and may hinder opportunities for at-home rehabilitation. Patients with lower MHI may face challenges accessing necessary equipment and resources, lack social support, and have limited transportation access for follow-up care, which may warrant consideration and longer planning of discharge to rehabilitation facilities.

While the role of household income as a determinant of health outcomes is evident in the U.S., similar patterns are observed globally, where lower income levels often correlate with reduced access to healthcare services and poorer health outcomes.^33,34^ This similarity across countries highlights how income universally impacts health, regardless of the specific healthcare system. Improving access to rehabilitation is recognized as a key factor in improving surgical outcomes globally. Ongoing research is highlighting the barriers to rehabilitation in low and middle-income countries and identifying areas needed for further study.^34-38^ While our study is conducted at a single medical center in the United States, it provides important insight into socioeconomic determinants of rehabilitation in a single model of healthcare and may guide further cost-value analyses of these dynamics globally.

Importantly, these findings come from a cohort of patients for whom care has already been established. While lack of income poses significant barriers to access to care, our findings come from patients who had already received spinal surgery at our medical center, providing further evidence of SDOH that exist beyond the initial barrier of access to care. By increasing discharge rates to rehabilitation and nursing facilities, poverty at the individual level may contribute to overall increased healthcare utilization and costs at the system level.^39,40^ Interventions providing patients the means and support to be discharged home safely for rehabilitation may prove cost-effective in the long run and improve patient satisfaction. Further study would be needed to determine if increasing coverage for at-home health care or access to transportation would save systemic costs of longer stays and rehabilitation facilities. Pre-surgical initiation of discharge coordination may identify potential barriers and inform immediate perioperative care to optimize rehabilitation.

### Limitations

While our study is novel in the isolation of a homogenous population and controlled evaluation of socioeconomic determinants of short-term surgical outcomes, our findings come with limitations. Our cohort comes from a retrospective analysis of patients at a single large academic center which limits our generalizability. In our CEM protocol, we limit our sample size by design in order to exactly match patients and isolate MHI as an independent variable. A limitation of CEM is the limited resolution of the control categories; for instance, by categorizing insurance into public and private, we may not capture variances within each group. Lower-income patients with private insurance may still have limited coverage for certain services, such as home health care, which may confound our results. Further, our study intentionally isolates MHI as the studied variable, though intricately related components of socioeconomic status such as education level, employment status, transportation barriers, and social support may also partially explain our results. This highlights the need for further research into composite measures of socioeconomic status and identifying which components are most sensitive to identify vulnerable patient populations who can benefit from risk-mitigation strategies.

The primary outcomes evaluated in this study are short-term surgical outcomes and do not provide information about long-term disease trajectories. These outcomes were deliberately chosen as they are easily interpreted, reproducible, and commonly used for quality assessment and improvement initiatives. These efforts should inform future study to capture longer-term dynamics of the social determinants of lumbar fusion outcomes. While MHI has historically been widely studied as a SDOH and is a broadly intuitive metric, income is only a single measurement to evaluate socioeconomic opportunity, and further study is warranted with other, more holistic metrics.

## Conclusion

This study isolated median household income as it relates to outcomes following lumbar fusion. Congruent with prior literature, we found significant univariate correlations when looking at MHI across all patients in our cohort. Between otherwise exactly matched patients, low-income individuals were significantly less likely to be discharged home than high-income counterparts and stayed in the hospital longer, yet no differences remained between surgical complications, ED visits, readmissions, or reoperations. Further studies must evaluate the mechanisms of these processes and target interventions to improve both patient satisfaction and healthcare costs.

## Appendix

MHI: Median Household Income
ED: Emergency Department
OR: Odds Ratio
SDOH: Social Determinants of Health
CEM: Coarsened exact matching
EHR: Electronic Health Record
BMI: Body mass index
ASA: American Society of Anesthesiologists
CCI: Charlson Comorbidity Index
